# Family resilience and neighborhood factors affect the association between digital media use and mental health among children: does sleep mediate the association?

**DOI:** 10.1007/s00431-023-04898-1

**Published:** 2023-03-16

**Authors:** Helal Uddin, Md. Khalid Hasan

**Affiliations:** 1 Department of Global Public Health, Karolinska Institutet, Solna, 17177 Sweden; 2 Department of Sociology, East West University, Dhaka, 1212 Bangladesh; 3 Unit for Research in Emergency and Disaster, Faculty of Medicine and Health Sciences, University of Oviedo, Oviedo, 33006 Spain; 4 Institute of Disaster Management and Vulnerability Studies, University of Dhaka, Dhaka 1000 Dhaka, Bangladesh

**Keywords:** Digital media use, Mental health, Sleep, Family resilience and connection, Neighborhood factors, USA

## Abstract

The associations between digital media use and mental well-being among children and adolescents have been inconclusive. We examined (i) the associations between digital media use and mental health outcomes, anxiety, depression, and ADHD, (ii) whether family resilience and neighborhood factors attenuate the associations, and (iii) whether sleep mediates these associations. We used the National Survey of Children’s Health data from 2019 to 2020. A total of 45,989 children’s (6–17 years) data were analyzed in this study. Multivariate logistic regression was used to assess the associations between digital media use and anxiety, depression, and ADHD. Path models and *Paramed* command in STATA were used to test the role of sleep as a mediator of these associations. The prevalence of heavy digital media users (who spent 4 or more hours per day) among the analytic sample was 30.52%, whereas anxiety was 13.81%, depression was 5.93%, and ADHD was 12.41%. Children in the heavy media user group had 63% increased odds of anxiety (95% CI: 1.32–2.01) and 99% increased odds of depression (95% CI: 1.35–2.94) after adjusting for sociodemographic factors, compared to the children in light media user group (who spent < 2 h per day), and these relations were significant at 0.01 level. However, family resilience and community factors significantly attenuated the effect of digital media use on anxiety and depression. Sleep did not mediate the associations between digital media use and anxiety or depression.

*Conclusions*: Family resilience and neighborhood factors protect against the harmful effects of digital media use. Further research is needed to examine the relationships of media contents, the presence of electronic devices in bedrooms, and sleep quality with mental health.

**Table:** 

**What is Known:**
• *Spending long hours on digital media may adversely affect children and adolescents' health and development. However, the mediating role of sleep in the association between digital media use and mental health outcomes is inconclusive.*
**What is New:**
• *Digital media use has detrimental effects on anxiety and depression. However, family resilience and neighborhood factors attenuated the association. The study highlights the importance of positive family functioning and neighborhood conditions reducing the harmful effects of digital media use.*

## Introduction

The use of electronic devices, including televisions, tablets, laptops, and smartphones, in the household is rapidly evolving and providing opportunities for extending the usage of digital media (e.g., social media use, texting, electronic gaming, and computer use) for both children and youth [1, 2]. Adolescents spent twofold more time on online platforms between 2006 and 2016 [3], as 95% have smartphones [3]. For example, a population-based study showed that US adolescents aged 14–17 years spent almost 4.59 h on the screen daily for recreational purposes [4] and teens (17–18 years) spent 6 h each day on online and social media [5]. Although appropriate digital media use benefits better communication, connectivity, and specific educational opportunities, the American Academy of Pediatrics (AAP) has suggested that children and youth (5–18 years) should not spend more than 2 h daily on digital media use [6]. Hence, spending long hours on digital media may adversely affect children and adolescents’ health and development [7].

Associations between digital media use and poor health outcomes, such as obesity [8], sleep problems [9], attention problems, hyperactivity, impaired cognition, and suicidal ideation [1], have been well documented. However, research exploring the association between screen-based digital media use and psychological well-being among children and adolescents has been inconclusive. While most studies have acknowledged the significant association between screen time spent on digital media and lower mental health [2, 10–12], some studies have documented null association [13, 14], negative association, or even benefits with higher screen time [15]. Thus, more research is required before concluding that digital media use is a predictor of children’s mental health outcomes since screen-based technologies are continuously evolving.

However, sleep duration may mediate the association between screen time spent on technological devices and mental health outcomes [16, 17], although the mediation role is less clear. For example, the AAP suggests 8 to 10 h of sleep duration each night for adolescents aged 13–18 years [18], but this period of sleep period may be disturbed by increased screen time use [19]. Consequently, the association between technologies and sleep is progressively discussed, as research shows that 50–90% of school-age children reported the problem of getting enough quality sleep [20]. In addition, adolescents who spend excessive time on screen-based media platforms can expose to sleep restriction and then suffer from shorter sleep durations [21] and poor sleep quality [9]. Furthermore, previous studies documented that screen time negatively impacts on sleep; in turn, inadequate sleep has been associated with depression [17] and anxiety [16]. Conversely, longitudinal studies showed a reciprocal relationship between screen use and sleep quality [22]; those with sleep problems gravitate to screens resulting in poorer sleep quality and duration [23].

While screen-based technology is adversely associated with the child’s health outcomes and development, protective factors can attenuate the adverse effects of the stressors on the outcomes. For example, stress-buffering models argue that healthy and positive family functioning and parental interaction can mitigate the harmful effects of stressful experiences on physical and mental health [24]. In addition, family resilience acts as a protective resource and significantly reduces the negative impact of adverse exposure on a child’s health and developmental outcomes [25, 26]. Furthermore, community or neighborhood-level physical and environmental factors such as social cohesion, including a sense of belongingness and safety [27], and the presence of amenities like parks, roads, sidewalks, and recreational centers [28] have a protective role in health, well-being, and development of children and adolescents and even promoting flourishing in many ways [26]. Theoretically, adolescents who experience parental restrictions (i.e., online security practices) on digital media use or positive parent–child relationships are less likely to be exposed to cyber victimization and detrimental experiences in the online platform [29–31], which eventually may impact their mental health. Similarly, a safe social environment and neighborhood amenities (i.e., recreational resources, parks, and playgrounds) are positively associated with increased physical activity participation and decreased screen-based activities, resulting in lower mental health symptoms [32–35]. However, although previous studies highlighted the role of protective factors in mitigating the harmful effect of adverse exposures, the empirical examination of whether household and neighborhood factors diminish the detrimental impact of excessive digital media use is absent from the literature.

Childhood is a critical period when developing and nurturing healthy social and emotional lifestyles are essential for psychological health and well-being. Using nationally representative cross-sectional data, the objectives of this study were to (1) examine the association between digital media use and mental health outcomes, (2) test whether family resilience and neighborhood factors attenuate the associations, and (3) examine whether sleep mediates the associations.

## Methods

### Study population

The study obtained data from the 2019 to 2020 National Survey of Children’s Health (NSCH). The NSCH, a nationally representative child-level household survey across the USA, collects data on various health and well-being indicators of children aged 0–17. The US Census Bureau conducts the survey. The NSCH collects data through phone interviews with parents or guardians selected through an address-based random sampling approach. The NSCH 2019–2020 combined dataset had 72,210 completed interviews (29,433 interviews for the NSCH 2019 and 42,777 interviews for the 2020 NSCH), of which 51,895 were for children aged 6–17 years. About 1.48% of parents/caregivers (*n* = 767) had missing information for digital media use, 1.49% (*n* = 774) had missing data for sleep, 2.69% (*n* = 1398) had missing information on neighborhood safety, 3.26% (*n* = 1692) had missing supportive neighborhood data, 3.04% (*n* = 1,578) had missing data on neighborhood amenities, 4.2% (*n* = 2,178) had missing family resilience information, 2.42% (*n* = 1,256) had missing family structure data, 1.17% (*n* = 605) had missing data on medication, and 1.61% (*n* = 836) had missing information on insurance type. We applied listwise deletion to create our final analytical sample of 45,989 children aged 6–17 years, with complete data on all study variables used in this study. As excluded missing cases may be a potential source of bias for explaining results, we tested the nature of missing cases, whether they were missing completely at random (MCAR) or not using Little’s MCAR test in STATA [36], before applying listwise deletion. Our analysis did not find the MCAR test as significant, suggesting that the missing cases were missing completely at random. Therefore, we did not continue with multiple imputations.

### Outcomes

#### Child’s anxiety

The NSCH 2019–2020 asked parents/caregivers whether “a doctor or other health care provider ever told you that this child has anxiety.” The responses were *do not have the condition*; *ever told, but do not currently have the condition*; and *currently have the condition*. A response of currently having the condition was considered a child with anxiety and coded 1. If the response was (i) child did not have the condition or (ii) ever told but does not currently have the condition, the child is considered without anxiety and coded 0.

#### Child’s depression

Parents/caregivers were asked whether “a doctor or other health care provider ever told you that this child has depression.” They were asked to select the following options: *do not have the condition*; *ever told, but do not currently have the condition*; and *currently have the condition*. Children were considered to meet the criteria for depression if they replied *currently to have the condition* and coded as 1. The rest of the responses were not supposed to meet the criteria and were coded 0.

#### Child’s ADHD

A child’s ADHD is measured based on parents/caregivers reporting a question about whether “a doctor or other health care provider ever told you that the child has attention-deficit disorder (ADD) or attention-deficit/hyperactivity disorder (ADHD).” The response options included *do not have the condition*; *ever told, but do not currently have the condition*; and *currently have the condition*. Therefore, we coded 0 for *do not have the condition* and *ever told, but do not currently have the condition*, and coded 1 for *currently have the condition*.

### Exposure

#### Digital media use

Digital media use was based on the parents/caregivers’ response to “on most weekdays, about how much time does this child usually spend in front of a television, computer, cellphone or other electronic device watching programs, playing games, accessing the internet or using social media (do not include time spent doing schoolwork).” The responses were less than 1 h, 1 h, 2 h, 3 h, and 4 or more hours. For the analysis, we created the following 3 categories: light user (who spent < 2 h), moderate user (who spent 2–3 h), and heavy user (who spent 4 or more hours). Remarkably, in our analytical sample (children aged 6–17 years), the heavy and light user categories are about the top and bottom quartile of the distribution for digital media use. In contrast, the moderate user category is approximately the middle 50% of the distribution. Even though children combined from moderate and heavy user categories overstep the maximum recommended hours per day in using digital media for children and youth [37–39], additionally they collectively represent a large majority of the NSCH 2019–2020 sample aged 6–17 years (81%) producing further divisions between these digital user categories both informative and necessary. Previous study also used the same categories of digital media use [40]. Additionally, for our mediation analysis, we recoded digital media use as a binary variable.

### Mediating variable

#### Sleep

The NSCH 2019–2020 measured adequate sleep by asking the respondents, “How many hours of sleep does the child get on average on most weeknights during the past week?” The responses were either recommended age-appropriate hours or less than recommended age-appropriate hours following the AAP sleep guidelines [41].

### Protective factors

#### Safe neighborhood

The NSCH 2019–2020 assessed neighborhood safety by asking respondents, “Does this child live in a safe neighborhood?” The possible response categories were (1) *definitely agree*, (2) *somewhat agree*, and (3) *somewhat or definitely disagree*. For the analysis, we recorded “yes” for *definitely agree* and “no” for *somewhat agree and somewhat or definitely disagree.* Previous study used similar conditions as safe neighborhoods [42]. 

#### Supportive neighborhood

The variable supportive neighborhood was measured by the following three items: (1) people in this neighborhood help each other out; (2) we watch out for each other’s children in this neighborhood; and (3) when we encounter difficulties, we know where to go for help in our community. Parents/caregivers were requested to choose to what extent they agreed with the statement from the following options: *definitely agree*, *somewhat agree*, *somewhat disagree*, or *definitely disagree*. The NSCH 2019–2020 calculated children in the supportive neighborhood if their parents/caregivers replied, “definitely agree” to at least one of the items above and “somewhat agree” or “definitely disagree” to the other two items.

#### Neighborhood amenities

The perceived neighborhood amenities were assessed by asking the question, “whether the child lives in a neighborhood that contains certain amenities—parks, recreation centers, sidewalks, or libraries.” For our analysis, we created a binary variable and measured “neighborhood amenity count” if the respondents answered “yes” for all four amenities.

#### Family resilience and connection index (FRCI)

We created the FRCI index using six parent–child bonding and connection items. Parents/caregivers were asked to assess how often family members were likely to do the following when the family is faced with problems: (1) “talk together about what to do,” (2) “work together to solve our problems,” (3) “know we have strengths to draw on,” and (4) “stay hopeful even in difficult times.” Moreover, parents/guardians were asked how well they (5) “can share ideas or talk about things that really matter” with their child and (6) are “handling the day-to-day demands of raising children.” For the first four items, the response options were *all of the time*, *most of the time*, and *none/some of the time*, and we coded them as 2, 1, and 0, respectively. For the last two questions, the responses were *very well*, *somewhat well*, and *not very well* or *not very well at all*, and we coded them as 2, 1, and 0, respectively. Finally, we summed the answers to all six items, creating a total FRCI score ranging between 0 and 12, with a higher score indicating better resilience and connection in the family. The internal consistency and reliability of the FRCI scale was very good (Cronbach’s alpha = 0.85) [25, 26].

### Confounders

Drawing on previous studies, we accounted for the following sociodemographic factors in the statistical analysis: child’s age (6–17 years: 6–10, 11–13, and 14–17); child’s sex (male or female); federal poverty level (0–99% FPL, 100–199% FPL, 200–399% FPL, and ≥ 400% FPL); parental education level (< high school, high school graduate, some college, and college graduate or more); insurance type (uninsured, public, private, and both public and private); child’s race/ethnicity (Hispanic, non-Hispanic Black, non-Hispanic White, and other); child’s nativity (US-born or foreign-born); primary language spoken at home (English or others); and family structure (single parents or two biological parents).

However, our analysis included the following comorbid (medical and behavioral) conditions: intellectual disability, cerebral palsy, autism, and behavioral or conduct problems, which could affect children’s day-to-day functioning and were associated with both their digital media use and well-being [2, 4]. Comorbid conditions were measured by asking the question: “whether a doctor or other healthcare provider ever told you that this child has the condition.” The responses were *do not have the condition*, *ever told, but no current condition*, and *currently have the condition*. A response to *currently have the condition* was regarded as the meeting criteria of comorbid conditions. Furthermore, medication for emotions/behavior/concentration issues and children with special healthcare needs were also included in our analysis. The response options were binary (yes, if they take medication).

### Statistical analysis

Using the chi-square test, we first presented the child’s characteristics through digital media use (Table 1). Next, we showed the prevalence of outcomes (anxiety, depression, and ADHD) by comorbid conditions, medication, and special healthcare needs (Table 2). We then used logistic regression analysis to get odds ratios (OR) and 95% CI for the associations of digital media use with mental health outcomes in separate models. Model 1 was unadjusted. Model 2 adjusted for age, sex, and survey year. Model 3 further included poverty level, parental education, and insurance type. Model 4 additionally adjusted for race, nativity, primary language at home, family structure, intellectual disability, cerebral palsy, autism, behavioral/conduct problems, medication, and special healthcare needs. Model 5 incorporated neighborhood-level factors (safe neighborhood, supportive neighborhood, and neighborhood amenities) with model 4. Model 6 fitted for the family resilience and connection index (FRCI) with model 4. Finally, model 7 adjusted for both neighborhood factors and FRCI with confounders (Table 3). Further, our analysis included an interaction term between digital media use and FRCI, safe neighborhood, supportive neighborhood, and neighborhood amenities for anxiety and depression outcomes. For ease of interpretation, we presented the marginal effects of digital media use from these interactions adjusted for all sociodemographic factors and comorbid conditions in Figs. 2 and 3.

**Table 1:** Sample characteristics by digital media use, NSCH 2019–2020 (*n* = 45,989)

**Attributes**	**Digital media use (hours per weekday)**						**Chi-square** ***p*** **value**
	***Light (8,366, 18.21%)***		***Moderate (23,557, 51.27%)***		***Heavy (14,022, 30.52%)***		
	n	%	n	%	n	%	
***Child’s characteristics***							
** Age, years**							
6–10	4759	29.55	8609	53.45	2738	17.0	< 0.001
11–13	1807	15.74	6147	53.54	3528	30.73	
14–17	1800	9.81	8801	47.94	7756	42.25	
Mean, SD	10.37, 3.37		11.91, 3.42		13.31, 3.05		
** Sex**							
Male	3989	16.81	12,155	51.23	7582	31.96	< 0.001
Female	4377	19.70	11,402	51.32	6440	28.98	
** Race**							
White, non-Hispanic	5993	19.06	16,496	52.46	8954	28.48	< 0.001
Black, non-Hispanic	418	14.06	1437	48.35	1117	37.58	
Hispanic	835	14.83	2839	50.41	1958	34.77	
Others/multi-racial	1120	18.99	2785	47.22	1993	33.79	
***Household characteristics***							
** Primary language at home**							
English	7869	18.25	22,187	51.47	13,053	30.28	< 0.001
Others	497	17.52	1370	48.31	969	34.17	
** Nativity**							
US-born	8074	18.27	22,739	51.45	13,380	30.28	< 0.001
Foreign-born	292	16.67	818	46.69	642	36.64	
** Family structure**							
Single parents	2147	14.37	7296	48.85	5493	36.78	< 0.001
Two biological parents	6198	20.06	16,198	52.44	8495	27.5	
***Socioeconomic status***							
** Household income, % FPL**^**¥**^							
0–99 FPL	902	17.89	2458	48.78	1683	33.37	< 0.001
100–199 FPL	1270	17.17	3714	50.20	2414	32.63	
200–399 FPL	2429	16.91	7405	51.56	4527	31.52	
≥ 400 FPL	3765	19.67	9980	52.13	5398	28.20	
** Highest education in household**							
< High school	182	16.34	532	47.76	400	35.91	< 0.001
High school	925	15.77	2953	50.35	1987	33.88	
Some college	1576	14.72	5393	50.37	3738	34.91	
College	5683	20.11	14,679	51.94	7897	27.95	
** Insurance type**							
Uninsured	427	19.81	1027	47.66	701	32.53	< 0.001
Public	1482	16.48	4411	49.04	3102	34.49	
Private	6172	18.69	17,259	52.27	9589	29.04	
Both public and private	285	16.06	860	48.45	630	35.49	
**Survey year**							
2019	4246	22.54	9989	53.03	4600	24.42	< 0.001
2020	4120	15.20	13,568	50.05	9422	34.75	

^¥^*FPL* federal poverty level

**Table 2:** Prevalence of parent-reported anxiety, depression, and ADHD among US children aged 6–17 years by medical/behavioral conditions, NSCH 2019–2020

**Characteristics**	***Anxiety***			***Depression***			***ADHD***		
	No (39,638, 86.19%)	Yes (6351, 13.81%)	Chi-square *p* value	No (43,263, 94.07%)	Yes (2726, 5.93%)	Chi-square* p* value	No (40,283, 87.59%)	Yes (5706, 12.41%)	Chi-square p value
	*n (%)*	*n (%)*		*n (%)*	*n *		*n (%)*	*n (%)*	
**Intellectual disability**									
No	39,332 (86.58)	6094 (13.42)	< 0.001	42,786 (94.19)	2640 (5.81)	< 0.001	39,967 (87.98)	5459 (12.02)	< 0.001
Yes	306 (54.35)	257 (45.65)		477 (84.72)	86 (15.28)		316 (56.13)	247 (43.87)	
**Cerebral palsy**									
No	39,530 (86.22)	6319 (13.78)	0.002	43,133 (94.08)	2716 (5.92)	0.542	40,169 (87.61)	5680 (12.39)	0.027
Yes	108 (77.14)	32 (22.86)		130 (92.86)	10 (7.14)		114 (81.43)	26 (18.57)	
**Autism**									
No	38,898 (87.51)	5551 (12.49)	< 0.001	42,031 (94.56)	2418 (5.44)	< 0.001	39,513 (88.9)	4936 (11.1)	< 0.001
Yes	740 (48.05)	800 (51.95)		1232 (80.0)	308 (20.0)		770 (50.0)	770 (50.0)	
**Behavioral problems**									
No	37,669 (89.33)	4501 (10.67)	< 0.001	40,383 (95.76)	1787 (4.24)	< 0.001	39,038 (92.57)	3132 (7.43)	< 0.001
Yes	1969 (51.56)	1850 (48.44)		2880 (75.41)	939 (24.59)		1245 (32.6)	2574 (67.4)	
**Medication**									
No	37,038 (91.97)	3235 (8.03)	< 0.001	39,315 (97.62)	958 (2.38)	< 0.001	38,596 (95.84)	1677 (4.16)	< 0.001
Yes	2600 (45.49)	3116 (54.51)		3948 (69.07)	1768 (30.93)		1687 (29.51)	4029 (70.49)	
**Special health care needs**									
No	31,404 (95.21)	1579 (4.79)	< 0.001	32,545 (98.67)	438 (1.33)	< 0.001	32,009 (97.05)	974 (2.95)	< 0.001
Yes	8234 (63.31)	4772 (36.69)		10,718 (82.41)	2288 (17.59)		8274 (63.62)	4732 (36.38)

**Table 3:** Adjusted association of digital media use with anxiety, depression, and ADHD, NSCH 2019–2020

**Outcomes**	**Model 1**		**Model 2**		**Model 3**		**Model 4**		**Model 5**		**Model 6**		**Model 7**	
	Crude model		Demographic model		Socioeconomic model		Sociodemographic model		Neighborhood factors		FRCI		Neighborhood + FRCI	
	OR	95% CI	OR	95% CI	OR	95% CI	OR	95% CI	OR	95% CI	OR	95% CI	OR	95% CI
**Anxiety** ***(light = Ref)***														
Moderate	1.43***	(1.20–1.71)	1.30***	(1.09–1.56)	1.30***	(1.08–1.55)	1.26**	(1.04–1.54)	1.25**	(1.03–1.52)	1.20*	(0.99–1.47)	1.20*	(0.99–1.46)
Heavy	2.40***	(2.00–2.88)	1.95***	(1.61–2.36)	1.93***	(1.59–2.34)	1.63***	(1.32–2.01)	1.59***	(1.29–1.97)	1.45***	(1.16–1.80)	1.45***	(1.16–1.80)
**Depression* (light = Ref)***														
Moderate	1.76***	(1.25–2.46)	1.44**	(1.02–2.03)	1.43**	(1.01–2.01)	1.27	(0.86–1.86)	1.25	(0.86–1.84)	1.19	(0.80–1.75)	1.19	(0.81–1.76)
Heavy	4.09***	(2.92–5.74)	2.70***	(1.89–3.87)	2.59***	(1.80–3.72)	1.99***	(1.35–2.94)	1.95***	(1.32–2.87)	1.71**	(1.13–2.59)	1.72***	(1.14–2.58)
**ADHD** ***(light = Ref)***														
Moderate	1.22**	(1.04–1.43)	1.11	(0.95–1.31)	1.10	(0.93–1.29)	1.02	(0.81–1.28)	1.02	(0.82–1.29)	1.01	(0.80–1.27)	1.01	(0.81–1.27)
Heavy	1.74***	(1.48–2.05)	1.51***	(1.27–1.81)	1.43***	(1.20–1.70)	0.98	(0.76–1.26)	0.98	(0.76–1.27)	0.96	(0.74–1.23)	0.96	(0.74–1.24)

*p ≤ 0.1, **p ≤ 0.05, ***p ≤ 0.01

Model 1: crude model

Model 2: model 1 + age, sex, and survey year

Model 3: model 2 + poverty, parental education, and insurance type

Model 4: model 3 + race, nativity, primary language, family structure, intellectual disability, cerebral palsy, autism, behavioral/conduct problems, medication, and special health care needs

Model 5: model 4 + neighborhood factors (safe neighborhood, supportive neighborhood, and neighborhood amenities)

Model 6: model 4 + family resilience and connection index (FRCI)

Model 7: model 4 + neighborhood factors, and FRCI

**Fig. 1:**
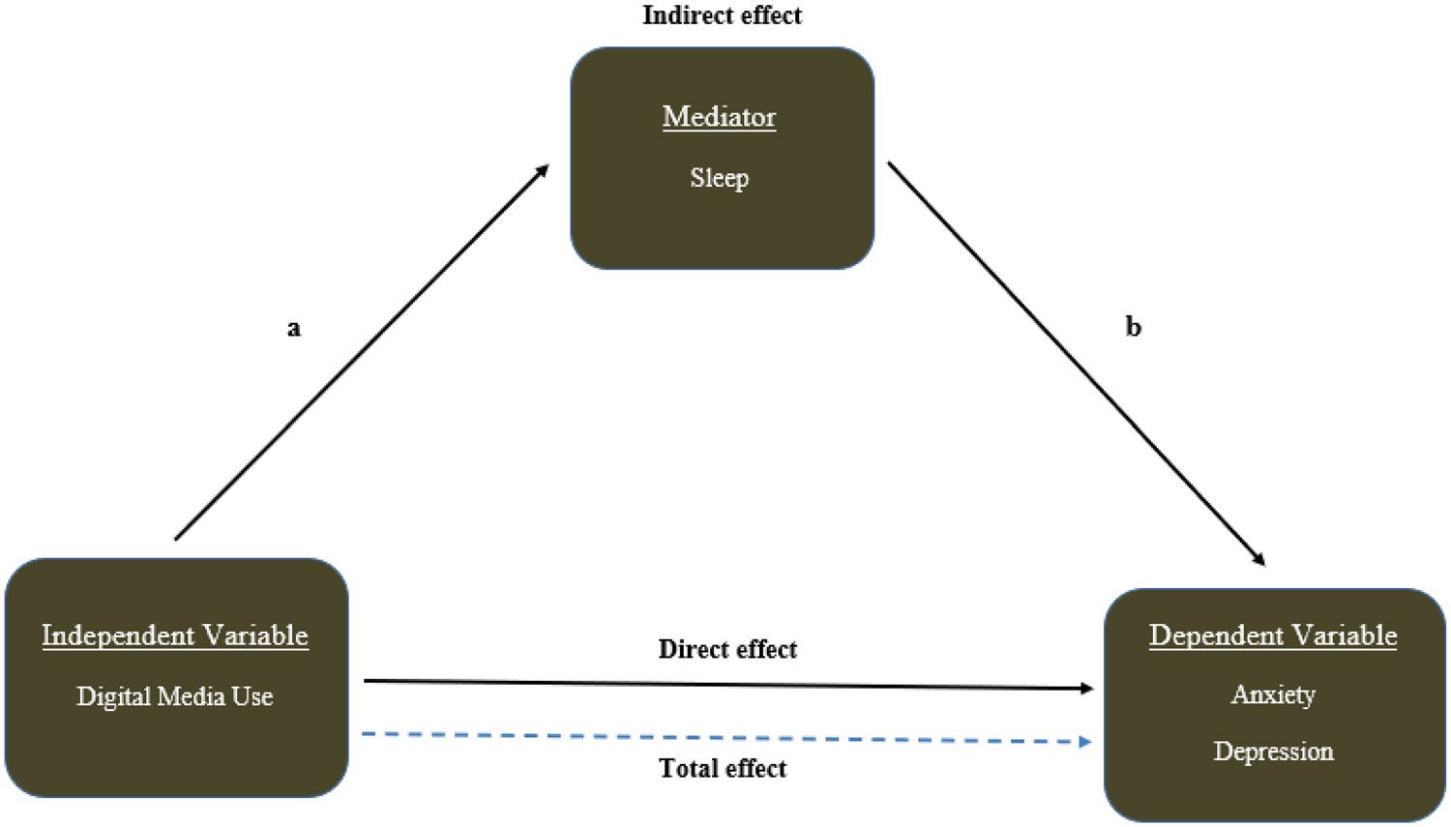
Mediation analysis. The figure represents the hypothesized relationship between digital media use, sleep, and anxiety/depression. **a** indicates the “a” path, and **b** indicates the “b” path of interaction. Analysis adjusted for confounders (not shown), including age, sex, federal poverty level, parental education, insurance type, child’s race/ethnicity, child’s nativity, primary language spoken at home, family structure, comorbid conditions, medication, special health care needs, and survey year

We conducted mediation analysis using path models to test the potential mediating role of sleep in the pathway between digital media use and mental health outcomes, adjusting for all confounders. Our analysis followed a two-step method to examine the mediation, which includes a model for (i) the mediator (i.e., sleep) conditional on the independent variable/predictor (i.e., digital media use) and covariates and (ii) the outcome variables (i.e., anxiety/depression) conditional on the independent variable/predictor (i.e., digital media use), the mediator (i.e., sleep), and covariates [43]. The *Paramed* command in STATA was used to estimate the mediating effects [44, 45]. Then the natural direct effects (nde), natural indirect effects (nie) (mediated by sleep), and the marginal total effects (mte) were calculated using the logit model applying the bias-corrected bootstrap 95% CI in 500 replications [45]. The proportion of mediated effects of sleep was estimated using the formula of (nie/[nie + nde]) [46]. Figure 1 presents the mediation model with digital media use as the predictor, sleep variable as the mediator, and anxiety/depression as the outcome. The *Paramed* command uses the counterfactual approach of mediation analysis and performs causal mediation following parametric regression models, which is the extension of the Baron and Kenny method [43]. To calculate the direct effect and indirect effects from the mediation model, we need the assumptions: (i) there are no unmeasured treatment (exposure)-outcome confounders given our covariates, (ii) there are no unmeasured mediator-outcome confounders given our covariates, (iii) there are no unmeasured treatment (exposure)-mediator confounders given our covariates, (iv) there is no effect of treatment (exposure) that confounds the mediator-outcome relationship [47, 48]. We used STATA version 17 (StataCorp L.P., College Station, TX) for all analyses.

## Results

Table 1 presents the descriptive statistics for sample characteristics by media use into three categories (light user, moderate user, and heavy user). The chi-square values showed that all sample characteristics were associated with digital media use. The prevalence of heavy media uses among children 6–17 years was 30.52%. In general, male, Black non-Hispanic, foreign-born, child with single parents, having both public and private insurance, child households with 0–99%FPL, and high school education had higher rates of heavy media exposure. For example, about 37% of the children with single parents were exposed to heavy media use compared to 27% of those with two biological parents.

Table 2 shows the prevalence of anxiety, depression, and ADHD by comorbid conditions. The prevalence of anxiety, depression, and ADHD among children aged 6–17 years in NSCH 2019–2020 was 13.81%, 5.93%, and 12.41%, respectively. In addition, 69.6% (*n* = 32,004) of children had no comorbidities, and only 30.4% (*n* = 13,985) had one or more conditions. However, more than half of children with anxiety had autism and took medication. More than a fifth of depressed children had autism, behavioral or conduct problems and took medication. Besides, more than two-thirds of children with ADHD had behavioral or conduct problems and took medication.

Table 3 displays odds ratios (OR) for the association between digital media use and mental health outcomes. Children with heavy digital media use were considerably more likely to have been diagnosed with anxiety, depression, or ADHD. In unadjusted analysis, children who were heavy digital media users were more likely to have anxiety than light or moderate users (OR = 2.40; 95% CI: 2.00–2.88). However, this association attenuated gradually after adjusting for sociodemographic variables and comorbidities in model 4 (OR = 1.63; 95% CI: 1.32–2.01). In model 5, when we further adjusted for neighborhood-level factors, including safe neighborhood, supportive neighborhood, and neighborhood amenities, the association between heavy digital media use and anxiety remained significant with OR attenuated a bit (OR = 1.59; 95% CI: 1.29–1.97). In model 6, the adjustment for FRCI further attenuated this association (OR = 1.45; 95% CI: 1.16–1.80). Finally, in the full model (model 7), the odds size of the association between heavy digital media use and anxiety decreased to 1.45 (*p* < 0.01) from 1.63 in model 4, a reduction of about 18%.

Moreover, in the crude model of depression, children with heavy digital use had higher odds (OR = 4.09; 95% CI: 2.92–5.74) of being depressed compared to light media users. In model 4, when we adjusted for sociodemographic factors and comorbidities, children who heavily use digital media had 2 times higher odds (95% CI: 1.35–2.94) of depression. After adjusting for the neighborhood factors in model 5 and FRCI in model 6, 4% and 28% odds were attenuated from model 4, respectively. In model 7, the association remained significant, with a 27% reduction of odds from model 4 (OR = 1.72; 95% CI: 1.14–1.58). Finally, in the unadjusted model of ADHD, children with heavy media exposure had a 1.74 times odds of having the condition. After further adjustment of sociodemographic factors, comorbidities, and protective factors in the following models (4, 5, 6, and 7), the association remained no longer significant (Table 3).

**Fig. 2:**
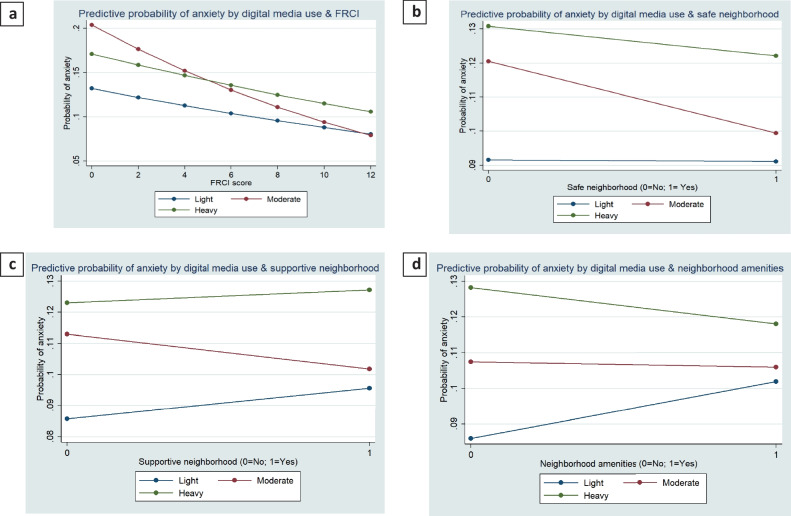
Predictive probability of anxiety by **a** digital media use*FRCI; **b** digital media use*safe neighborhood; **c** digital media use*supportive neighborhood; **d** digital media use*neighborhood amenities

**Fig. 3:**
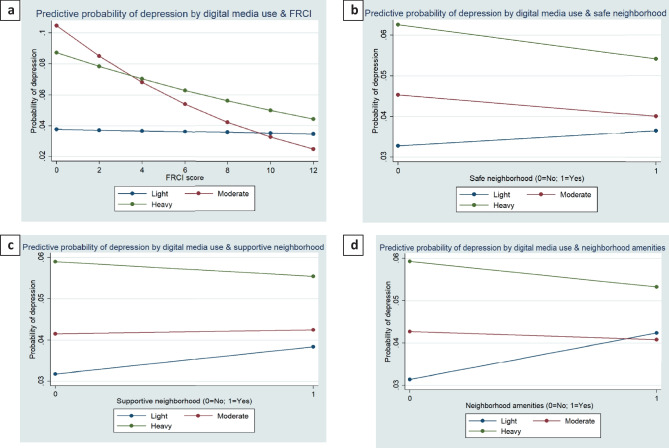
Predictive probability of depression by **a** digital media use*FRCI; **b** digital media use*safe neighborhood; **c** digital media use*supportive neighborhood; **d** digital media use*neighborhood amenities

We tested the interactions between digital media use, FRCI, and neighborhood-level factors in the sociodemograhic model (model 4) for anxiety and depression. The interaction between digital media use, FRCI, and three neighborhood factors was significant (*p* ≤ 0.01) for anxiety and depression outcomes. The average marginal effects of digital media use on reporting anxiety and depression by the level of family resilience and neighborhood factors were presented in Figs. 2 and 3. The marginal effects of digital media use on two mental health outcomes indicated that the effects of digital media use on anxiety and depression outcomes vary by the score of FRCI and neighborhood conditions (i.e., safe neighborhood, supportive neighborhood, and neighborhood amenities). In sum, the effects of digital media use on anxiety and depression were weakened with the higher score of FRCI and the presence of positive neighborhood conditions.

Figure 1 shows the complete models with sleep as the mediating variable, digital media use as the predictor, and anxiety/depression as the outcome variable. For the anxiety outcome, digital media use was significantly associated with adequate hours of sleep (path a), and adequate hours of sleep was significantly associated with anxiety (path b) (result not present). Similarly, for the depression outcome, digital media use was significantly associated with sleep (path a), and sleep was significantly associated with depression (path b). Table 4 shows the direct, indirect, and total effects of digital media use on anxiety and depression outcomes using mediation analysis. Both the total effects and direct effects were statistically significant (OR = 1.343; 95% CI: 1.25–1.43) and (OR = 1.335; 95% CI: 1.25–1.42), respectively, with *p* ≤ 0.001 for anxiety outcome, while the indirect effects were not significant (OR = 1.005; 95% CI: 1.00 – 1.01; *p* = 0.723). The proportion of the effects of digital media use mediated by sleep on anxiety was 43% (nie/[nie + nde] × 100). A similar pattern was also found for the depression outcome. The total and direct effects were significant, whereas the indirect effects were not significant. The proportion of the effects of digital media use mediated by sleep on depression was 40%. Therefore, sleep did not mediate the association of digital media use with anxiety or depression.

**Table 4:** Mediation analysis of the association between digital media use and anxiety and depression by sleep^¥^, NSCH 2019–2020

**Outcome variables**	**Digital media use (light use = Ref)**			
	OR	Std Err	95% CI	p value
** Anxiety**				
Natural direct effect	1.335***	0.044	1.25–1.42	< 0.001
Natural indirect effect	1.005	0.002	1.00–1.01	0.723
Marginal total effect	1.343***	0.044	1.25–1.43	< 0.001
Proportion of mediation	43%			
** Depression**				
Natural direct effect	1.548***	0.08	1.40–1.71	< 0.001
Natural indirect effect	1.014	0.003	1.01–1.02	0.402
Marginal total effect	1.570***	0.081	1.41–1.72	< 0.001
Proportion of mediation	40%			

*OR*  odds ratio, *Std Err* standard error, *CI* confidence interval

^¥^Each model adjusted for age, sex, poverty level, parental education, insurance type, race, nativity, primary language at home, family structure, intellectual disability, cerebral palsy, autism, behavioral/conduct problems, medication, special health care needs, and survey year

***p ≤ 0.001

## Discussion

As the likelihood of spending too much time using digital media has become a growing concern among public health experts, psychologists, physicians, and parents, research arguing the associations between digital media use and children’s well-being is critically needed. Our primary aim in this study was to examine the mitigating role of family resilience and community-level factors in the pathway between digital media use and children’s mental health outcomes, as well as test whether sleep mediates the associations. In this large representative sample of NSCH 2019–2020, 30% of children were heavy digital media users (4 or more hours per weekday), 14% had anxiety, 6% had depression, and 12% had ADHD. The findings indicate that the associations of digital media use with anxiety and depression were statistically significant, except for ADHD. In addition, family and neighborhood factors significantly attenuated the association between digital media use and anxiety and depression. Finally, the mediation analysis showed that sleep did not mediate the association of digital media use with anxiety and depression.

### Association between digital media use and children’s mental health

Psychosocial reasons support our findings on digital media use and its adverse psychological health outcomes. For example, children with light use of digital media may benefit from devices spending their time consciously and enjoying more positive and protective content. Conversely, increased digital media use may replace healthy habits like adequate sleeping, reading, social interaction, and physical activity [2], resulting in a higher risk for mental health problems. In addition, adolescents who spend more time on screen may risk being self-isolated [49] and may expose to more harmful content like cyberbullying [50], leading to a higher likelihood of anxiety and depression [4]. However, longitudinal studies argued that screen time for digital media and sleep predicts poorer mental health [10, 16], but few studies showed a bidirectional association of screen time with sleep, anxiety, and depression [51]. Hence, in line with previous studies, our findings support the hypothesis that increased time in using digital media predicts anxiety and depression [2, 4, 10, 12].

However, our analysis found a null association between digital media use and ADHD in a fully adjusted sociodemographic model among children, although previous studies reported a significant association between screen time and self-reported ADHD among older adults (at least 18 years or older) [52]. Additionally, some empirical studies indicated bidirectional association and showed ADHD symptoms as a predictor for excessive media use, particularly among adults [53–55]. However, a meta-analysis found a small significant association between media use and ADHD-related behavioral aspect for children and adolescents (*r* = 0.12), while they emphasized media content (i.e., violent and fast pace content) rather than overall media use [56]. For example, violent media content is related to poor self-control and increased arousal level, which hampers self-regulation, resulting in attention problems [57]. Additionally, poor quality of sleep due to sympathetic arousal level may work as a potential mechanism that shows hyperactivity and sensational seeking through overtiredness and consequently trigger ADHD-related behaviors [58]. Hence, findings are mixed for the association between digital media use and ADHD as well as digital media use may be associated with ADHD-related symptoms through several pathways. More studies are required to explore the direction of the association and the underlying mechanism of how digital media use is associated with ADHD, particularly for children.

### Protective role of family resilience and neighborhood factors and digital media use

We conceptualized that family resilience and connection and neighborhood factors would mitigate the harmful effect of digital media use on children’s mental health. The analysis found a significant reduction in the odds of digital media use. Previous studies also support the findings [25, 31, 59]. For example, a cross-sectional study using school-going adolescents in Bermuda showed that strong parental relationships appear more protective against harmful content on digital media, like cyberbullying victimization [31, 59]. In addition, studies found that a higher level of family resilience mitigates the detrimental effects of adverse exposures in childhood on mental health [25]. Further, neighborhood-level factors such as poorer access to outdoor activities [59] and more insufficient perceived esthetics [28] were associated with higher screen time on devices [60]. Thus, the findings of our study support the empirical evidence that family and social conditions such as family relationships and neighborhood characteristics of the children are considered in existing intervention schemes.

### Mediating role of sleep between digital media use and children’s mental health

We hypothesized that sleep mediates the pathway between digital media use and children’s mental health outcomes. Unlike previous studies [19, 49], the mediation analysis showed that sleep did not mediate the association, which is supported by another recent study [2]. However, in line with previous studies, significant associations remain between screen time in digital media use and adequate sleep hours [2, 61] as well as adequate sleep hours with anxiety and depression [62, 63]. Other factors (i.e., what times during the day engaged in digital media use) may have a crucial role in predicting adequate sleep duration. For example, screen use before bedtime is associated with poor sleep quality and delayed bedtimes [14, 19]. Moreover, excessive time on screen-based media platforms hampers sleep durations [21] and sleep quality [9]. Hence, further research is needed focusing on digital media using time and content in relation to bedtime and sleep quality. Besides, sleep duration may have a reciprocal association with mental health conditions [63], which requires longitudinal studies to assess these associations. Therefore, the associations of digital media use with mental health outcomes among children and adolescents are complex, and other variables may mediate the association, as the previous studies reported sleep partially mediate the associations, including sleep onset difficulties and sleep quality in their analytic models [16, 64].

The study is not beyond limitations. Firstly, the variables used in this analysis were parent-/caregiver-reported, which may present bias, including underestimating the number of hours spent on digital media use. Secondly, the NSCH measured anxiety, depression, and ADHD by asking caregivers whether a healthcare provider ever told them that the child had the condition. It is not clear how the child was diagnosed with the conditions. In addition, parents may underreport the symptoms of the conditions to the healthcare providers for existing social stigma on mental health, leading to misdiagnosis and underreporting of the NSCH. Thirdly, being a cross-sectional study, it is impossible to calculate the causal relationship between the variables. Despite these limitations, one of the key strengths of this study is to use of nationally representative data, which permits us to infer the result at the national level. In addition, our analysis incorporates both family and community-level factors to examine the protective role against digital media use. Hence, cChildhood is a vulnerable period of development in the life course and has a risk for anxiety and depression because of their social surroundings; further research is needed to examine the relationships of media contents, the presence of electronic devices in bedrooms, and sleep quality, as they relate to anxiety and depression.

## Conclusions

This study supported that increased time for digital media use may contribute to poorer psychological health outcomes. In addition, family resilience and community factors significantly attenuate the effect of digital media use on anxiety and depression. These findings highlight the importance of positive family functioning, neighborhood conditions, and recommended sleep hours and suggest the need for policies and interventions to mitigate the detrimental effect of heavy digital media use. Therefore, our findings support the US Department of Education’s mission to ensure a successful educational environment for children and adolescents by providing more resources for families, communities, and educational institutions. For example, state, federal, and local agencies constantly work to ensure children’s better health and well-being, particularly for children with different physical and mental challenges [65, 66]. The Administration on Children and Families and Integrated Care for Kids Model involve some concerned organizations that are working actively to improve the health and well-being of US children [66]. Hence, child protection agencies and responsible organizations should consider intervention programs, including improving economic and noneconomic resources to strengthen family cohesion and parent–child connections and neighborhood environments, as well as educating programs for both children and their family members about the negative effect of excessive screen time on sleep and mental health [2].

## Data Availability

The datasets generated and/or analyzed during the current study are available in the National Survey of Children’s Health (NSCH) (https://www.childhealthdata.org/dataset).

## Abbreviations

AAP: American Academy of Pediatrics
ADHD: Attention- deficit/hyperactivity disorder
FPL: Federal Poverty level
FRCI: Family resilience and connection index
NSCH: National Survey of Children’s Health
